# The effect of lipid-lowering therapy on lipid-related residual risk factors: a prospective study

**DOI:** 10.1186/s12944-024-02078-0

**Published:** 2024-05-07

**Authors:** Zhifan Li, Yanan Gao, Qianhong Lu, Zheng Yin, Shuang Zhang, Wenjia Zhang, Yonggang Sui, Yanlu Xu, Jianjun Li, Kefei Dou, Jie Qian, Hong Qiu, Naqiong Wu

**Affiliations:** Cardiometabolic Center, Fuwai Hospital, National Center for Cardiovascular Diseases, Chinese Academy of Medical Sciences & Peking Union Medical College, Beijing, 100037 China

**Keywords:** Remnant cholesterol, Nonhigh-density lipoprotein cholesterol, Lipid-lowering therapy, Atherosclerotic cardiovascular disease, Low-density lipoprotein cholesterol

## Abstract

**Background:**

Remnant cholesterol (RC) and nonhigh-density lipoprotein cholesterol (nonHDL-C) are key risk factors for atherosclerotic cardiovascular disease (ASCVD), with apolipoprotein B (apoB) and lipoprotein(a) [Lp(a)] also contributing to its residual risk. However, real-world population-based evidence regarding the impact of current clinical LDL-C-centric lipid-lowering therapy (LLT) on achieving RC and nonHDL-C goals, as well as on modifying residual CVD risk factors is limited.

**Methods:**

This prospective observational study enrolled 897 CVD patients from September, 2020 to July, 2021. All participants had previously received low-/moderate-intensity LLT and were discharged with either low-/moderate-intensity LLT or high-intensity LLT. After a median follow-up of 3 months, changes in RC, nonHDL-C, and other biomarkers were assessed. Multivariate logistic regression was performed to analyze the impact of the LLT on goal attainment.

**Results:**

Among all patients, 83.50% transitioned to high-intensity LLT from low or moderate. After follow-up, the high-intensity group saw significantly greater reductions in RC (-20.51% vs. -3.90%, *P* = 0.025), nonHDL-C (-25.12% vs. 0.00%, *P* < 0.001), apoB (-19.35% vs. -3.17%, *P* < 0.001), triglycerides (-17.82% vs. -6.62%, *P* < 0.001), and LDL-C and total cholesterol. Spearman correlation analysis revealed that LDL-C reduction from current LLT was strongly correlated with nonHDL-C reduction (*r* = 0.87, *P* < 0.001). Patients who received high-intensity LLT had significant improvements in attainment of RC (from 44.2% to 60.7%, χ² = 39.23, *P* < 0.001) and nonHDL-C (from 19.4% to 56.9%, χ² = 226.06, *P* < 0.001) goals. Furthermore, multivariate logistic regression showed that high-intensity LLT was a protective factor for RC [odds ratio (OR) = 0.66; 95% confidence intervals (CI), 0.45–0.97; *P* = 0.033] and nonHDL-C goal attainment (OR = 0.51; 95% CI, 0.34–0.75; *P* < 0.001), without a significant increase of adverse reactions.

**Conclusion:**

Current levels of clinically prescribed LDL-C-centric treatment can reduce RC and other lipid-related residual risk factors, but high-intensity LLT is better at achieving nonHDL-C and RC goals than low-/moderate-intensity LLT, with a good safety profile. More targeted RC treatments are still needed to reduce residual lipid risk further.

**Supplementary Information:**

The online version contains supplementary material available at 10.1186/s12944-024-02078-0.

## Introduction

Lipid-lowering therapy (LLT) is effective in reducing atherosclerotic cardiovascular disease (ASCVD) risk by targeting low-density lipoprotein cholesterol (LDL-C). Statins remain the mainstay of LLT, though ezetimibe, proprotein convertase subtilisin/kexin type 9 inhibitors (PCSK9i) and bempedoic acid are effective non-statin options recommended by current treatment guidelines [1–3]. However, despite widespread use of LDL-C-centric LLT, residual “lipid-associated” cardiovascular risk remains a problem, with a considerable number of predicted ASCVD unable to be averted [4].

Remnant cholesterol (RC), which encompasses the cholesterol content carried in triglyceride (TG)-rich lipoproteins, is a causal risk factor for ASCVD [5]. Using a broader definition, RC can include cholesterol of intermediate-density lipoprotein, very low-density lipoprotein (VLDL) and VLDL remnants, and chylomicron remnants [6]. It can be directly measured or simply calculated as total cholesterol (TC) minus LDL-C minus high-density lipoprotein cholesterol (HDL-C) [7]. A large prospective cohort study from the Danish general population showed that high RC was associated with increased risk of cardiovascular death, ischemic stroke, myocardial infarction (MI), aortic stenosis, peripheral artery disease (PAD) and all-cause mortality compared to participants with RC < 0.5 mmol/L (< 19 mg/dL) [8–11].

NonHDL comprises pro-atherogenic lipoproteins, nearly all of which contain pro-atherogenic apolipoprotein B (apoB). This type of cholesterol content (nonHDL-C) can be calculated by adding LDL-C to RC or subtracting HDL-C from TC [12]. Currently, aggregate findings suggest that nonHDL-C and apoB are strong predictors of ASCVD, and many guidelines recommend that they be used as secondary LLT targets or even primary targets to reduce residual risk [1, 2, 13, 14]. Lipoprotein(a) (Lp[a]), which is composed of LDL-like part, apoB-100 and apolipoprotein(a), is also one of the residual risk factors for ASCVD even with successful LDL-C reduction [15]. Therefore, in clinical trials of new LLTs, the impact of the treatments on these indicators has also become a focus.

However, real-world population-based evidence regarding the relationships between the achievement of RC and nonHDL-C goals, managing residual cardiovascular risk, and current clinical LLT is limited. It is hypothesized that clinical LDL-C-centric LLT impacts other residual risk-related lipid profiles, particularly RC and nonHDL-C, and that it indeed helps in achieving these respective treatment goals. This study provides real-world evidence on the efficacy of current LLT and specifically investigates the effects of transitioning from low-/moderate-intensity to high-intensity LLT.

## Methods

### Study participants

**Fig. 1:**
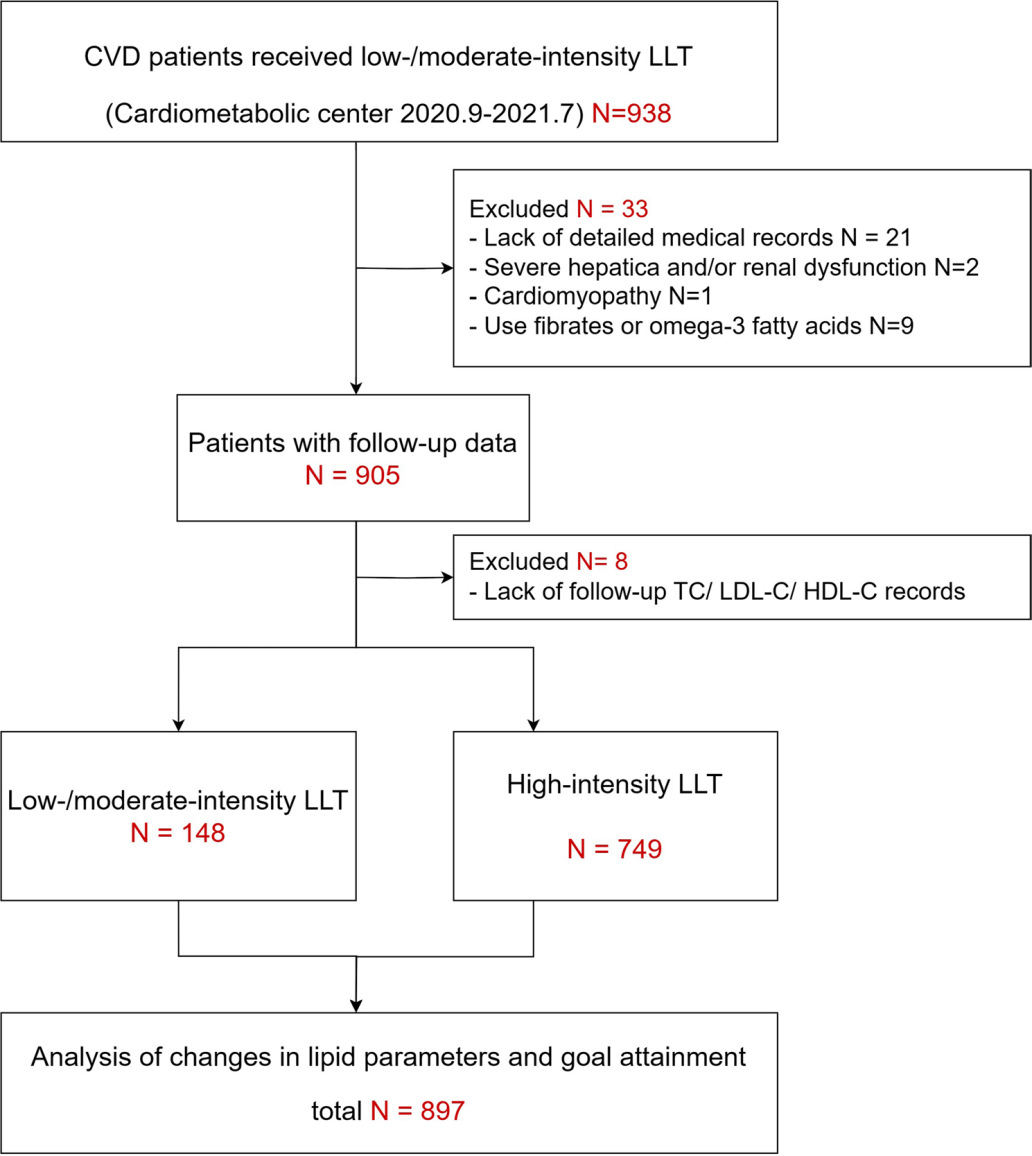
Flowchart of study participant selection. Note: CVD, cardiovascular disease; LLT, lipid-lowering therapy; TC, total cholesterol; LDL-C, low-density lipoprotein cholesterol; HDL-C, high-density lipoprotein cholesterol

This study was based on a prospective, single-center, observational cohort of CVD patients consecutively admitted to Fuwai hospital in North China between September 1, 2020 and July 31, 2021. Patients were only included if they had clinically evident CVD (defined as recent acute coronary syndrome (< 1 year), previous MI, stable or unstable angina, previous revascularization in coronary or other large and medium-sized arteries, history of ischemic stroke, symptomatic PAD with evidence of atherosclerotic origin, or coronary angiography showing ≥ 50% stenosis in at least one major coronary artery), and were on background low-/moderate statin monotherapy (atorvastatin 10 mg/d or 20 mg/d, rosuvastatin 10 mg/d, pitavastatin 2 mg/d or 4 mg/d, pravastatin 40 mg/d, fluvastatin 80 mg/d) or ezetimibe (10 mg/d) monotherapy, and were discharged with either low-/moderate-intensity LLT or high-intensity LLT (statin plus ezetimibe or PCSK9i, rosuvastatin 20 mg/d or atorvastatin 40 mg/d monotherapy) [16]. Potential patients were excluded if they had any of the following: critical lack of baseline medical records or examination data, severe hepatic and/or renal dysfunction, cardiomyopathy, severe blood system disease, or malignancies. Additionally, any patient who was taking fibrates or omega-3 fatty acids was also excluded due to their known TG-lowering effects. In the end, 897 patients were enrolled (Fig. 1), and written informed consent was received from all participants prior to enrollment. This study was approved by Fuwai hospital’s ethics committee and followed the Declaration of Helsinki.

### Data collection

Experienced clinical personnel assessed weight, height, pulse, systolic and diastolic blood pressure (SBP and DBP), and echocardiography. Past history and medication history were also recorded based on medical records and self-reports. Blood samples were drawn after fasting overnight to measure lipid profiles and routine blood chemistry using an automatic biochemistry analyzer at the hospital’s central chemistry laboratory. LDL-C was primarily calculated using the Friedwald formula, with direct measurement employed in cases of significantly elevated TG. NonHDL-C was estimated by subtracting HDL-C from TC, and RC was determined as TC– HDL-C– LDL-C. The choice of LLT and other medications at discharge was at the discretion of the clinicians. All data were collected by investigators blinded to this study.

### Endpoints

Follow-up data were obtained through patient medical records during subsequent visits and through regular contact via phone or messaging by an independent follow-up team, and lipid profiles and LLT strategies were recorded in detail. The endpoints of interest in this study were the change in lipid profiles (LDL-C, RC, nonHDL-C, TG, TC, HDL-C, Lp(a), apoA1 and apoB) and RC and nonHDL-C goal achievement. According to the 2019 ESC/EAS guidelines for very-high-risk ASCVD patients, the goal for nonHDL-C is < 2.2 mmol/L [1]. However, since the current guidelines have not yet set a target for RC, a threshold of 0.5 mmol/L was used based on large population studies [10]. Changes in hepatic and renal function indicators and related adverse reactions [aspartate aminotransferase (AST) > 120 IU/L, and/or alanine aminotransferase (ALT) > 150 IU/L] were also assessed.

### Statistical methods

Categorical variables were expressed as percentages (%) and continuous variables as means (± standard deviation, SD) or medians (range) for normal and nonnormal variables, respectively, after their distributions were evaluated using the Shapiro-Wilk test. Comparisons between groups of continuous variables were made using Student’s *t*-test or the Wilcoxon rank-sum test, and categorical variables were compared using the chi-squared test or Fisher’s exact test according to the normality of the variables. Scatter plots, Spearman’s rank correlation coefficients, and correlated *P*-values were generated for the percentage changes in LDL-C follow-up relative to baseline with percentage changes during follow-up in nonHDL-C, apoB, TGs, and remnant cholesterol for total participants and within each group (low-/moderate-intensity LLT and high-intensity LLT) as well. Univariate and multivariate logistic regressions were carried out to assess the impacts of the variables on goal attainment of RC and nonHDL-C in terms of odds ratio (OR) and 95% confidence intervals (CIs). LLT, age, sex, BMI, SBP, baseline levels of LDL-C and RC or nonHDL-C, history of PCI, ACS, hypertension, DM, and smoking status were included in the adjusted models. In the sensitivity analysis, an exploration of potential interactions between age, sex, and treatment regimens was undertaken. Interaction terms for age and sex were incorporated individually into the statistical model to assess their potential impact on the treatment outcomes. If significant interactions were to arise, the results were carefully outlined and reported for subgroups stratified by age (< 60/≥60) and sex (male/female). All statistical analyses were performed in R software version 4.1.1 (R Foundation for Statistical Computing, Vienna, Austria), and all reported probability values were two-sided, with *P* < 0.05 threshold for statistical significance.

## Results

### Clinical characteristics at baseline

Of the 897 participants were recruited for this study, 749 patients were adjusted to high-intensity LLT, and 148 patients continued with low-/moderate-intensity LLT according to their physicians’ decisions. In the high-intensity LLT group, 95.86% were using statins along with ezetimibe, and 30 individuals were on PCSK9i (detailed medications are recorded in Supplemental Table 1). Baseline demographic and clinical characteristics are reported in Table 1 (lipid profiles are shown in Table 2). Briefly, compared to continuing low-/moderate-intensity LLT, the patients in the high-intensity LLT group were younger, with higher levels of atherogenic lipids and high-sensitivity C-reactive protein, and a higher proportion of coronary artery bypass graft history, smoking history, and clopidogrel use.

**Table 1:** Baseline characteristics of the study population according to LLT type at discharge

Variables	Total (*N* = 897)	Low-/moderate-intensity LLT (*N* = 148)	High-intensity LLT (*N* = 749)	P value
Male, n (%)	673 (75.03%)	106 (71.6%)	567 (75.7%)	0.345
Age, years	58.48 ± 9.98	60.24 ± 9.91	58.14 ± 9.96	**0.019**
BMI, kg/m^2^	26.08 ± 3.12	25.82 ± 3.42	26.13 ± 3.06	0.270
SBP, mmHg	136.86 ± 17.27	136.00 ± 17.05	137.03 ± 17.32	0.511
DBP, mmHg	78.87 ± 10.52	77.86 ± 9.78	79.07 ± 10.66	0.201
ACS, n (%)	780 (86.96%)	125 (84.5%)	655 (87.4%)	0.393
PCI history, n (%)	251 (28.30%)	45 (30.6%)	206 (27.8%)	0.561
CABG history, n (%)	11 (1.24%)	5 (3.4%)	6 (0.8%)	**0.029**
Stroke, n (%)	51 (5.69%)	7 (4.7%)	44 (5.9%)	0.720
Hypertension, n (%)	545 (60.76%)	94 (63.5%)	451 (60.2%)	0.510
Hyperlipidemia, n (%)	614 (68.45%)	109 (73.6%)	505 (67.4%)	0.164
Diabetes mellitus, n (%)	268 (29.88%)	53 (35.8%)	215 (28.7%)	0.104
Smoking history, n (%)	450 (50.17%)	61 (41.2%)	389 (51.9%)	**0.022**
LVEDD, mm	47.00 (44.00 to 50.00)	47.00 (44.00 to 51.00)	47.00 (44.00 to 50.00)	0.496
LVEF, %	63.00 (60.00 to 65.00)	63.00 (60.00 to 65.00)	63.00 (60.00 to 65.00)	0.367
Hs-CRP, mg/L	0.98 (0.47 to 2.29)	0.81 (0.37 to 1.88)	1.01 (0.49 to 2.40)	**0.009**
Medications at discharge				
Aspirin, n (%)	844 (94.09%)	134 (90.5%)	710 (94.8%)	0.070
Clopidogrel, n (%)	449 (50.06%)	62 (41.9%)	387 (51.7%)	**0.037**
β-blockers, n (%)	681 (75.92%)	113 (76.4%)	568 (75.8%)	0.977
Nitrate, n (%)	792 (88.29%)	128 (86.5%)	664 (88.7%)	0.543
CCB, n (%)	540 (60.20%)	85 (57.4%)	455 (60.7%)	0.509
ACEI, n (%)	52 (5.80%)	3 (2%)	49 (6.6%)	0.050
ARB, n (%)	107 (11.94%)	20 (13.5%)	87 (11.6%)	0.738
Diuretic, n (%)	67 (7.48%)	17 (11.5%)	50 (6.7%)	0.063

*Note* Data are expressed as mean ± SD, median (Q1, Q3), or number (%). LLT, lipid-lowering therapy; BMI, body mass index; SBP, systolic blood pressure; DBP, diastolic blood pressure; ACS, acute coronary syndrome; PCI, percutaneous coronary intervention; CABG, coronary artery bypass graft; LVEDD, left ventricular end diastolic diameter; LVEF, left ventricular ejection fraction; Hs-CRP, high-sensitivity C-reactive protein; CCB, calcium channel blockers; ACEI, angiotensin converting enzyme inhibitors; ARB, angiotensin II receptor blockers

**Table 2:** Effects of LLT on lipid profiles

Variables	Total (*N* = 897)	Low-/moderate-intensity LLT (*N* = 148)	High-intensity LLT (*N* = 749)	P value
**LDL-C, measured**				
Baseline, mmol/L	2.15 (1.70 to 2.76)	1.72 (1.35 to 2.26)	2.22 (1.82 to 2.82)	**< 0.001**
Follow-up, mmol/L	1.62 (1.29 to 2.09)	1.79 (1.33 to 2.22)	1.60 (1.27 to 2.07)	**0.016**
Median change from baseline — %	-23.82 (-42.23 to 1.33)	-1.56 (-20.88 to 22.38)	-27.40 (-44.87 to -4.84)	**< 0.001**
**RC, calculated**				
Baseline, mmol/L	0.51 (0.39 to 0.68)	0.46 (0.33 to 0.63)	0.52 (0.40 to 0.69)	**< 0.001**
Follow-up, mmol/L	0.44 (0.29 to 0.63)	0.44 (0.29 to 0.59)	0.43 (0.30 to 0.58)	0.985
Median change from baseline — %	-18.18 (-46.15 to 27.59)	-3.90 (-43.68 to 50.00)	-20.51 (-46.67 to 23.53)	**0.025**
**NonHDL-C, calculated**				
Baseline, mmol/L	2.73 (2.20 to 3.43)	2.14 (1.79 to 3.03)	2.79 (2.32 to 3.49)	**< 0.001**
Follow-up, mmol/L	2.10 (1.68 to 2.62)	2.12 (1.66 to 2.56)	2.00 (1.59 to 2.48)	0.336
Median change from baseline — %	-22.55 (-39.93 to -0.45)	0.00 (-21.27 to 18.73)	-25.12 (-41.95 to -5.34)	**< 0.001**
**TG, measured**				
Baseline, mmol/L	1.48 (1.08 to 2.09)	1.29 (0.98 to 1.90)	1.51 (1.11 to 2.12)	**0.005**
Follow-up, mmol/L	1.20 (0.88 to 1.70)	1.21 (0.90 to 1.69)	1.18 (0.88 to 1.65)	0.853
Median change from baseline — %	-15.94 (-38.87 to 12.15)	-6.62 (-31.51 to 25.23)	-17.82 (-40.25 to 8.11)	**< 0.001**
**TC, measured**				
Baseline, mmol/L	3.88 (3.29 to 4.60)	3.39 (2.96 to 4.14)	3.95 (3.41 to 4.68)	**< 0.001**
Follow-up, mmol/L	3.24 (2.76 to 3.84)	3.23 (2.81 to 3.76)	3.10 (2.65 to 3.61)	0.219
Median change from baseline — %	-15.64 (-30.26 to 0.98)	0.08 (-13.87 to 12.18)	-18.14 (-31.55 to -3.70)	**< 0.001**
**HDL-C, measured**				
Baseline, mmol/L	1.11 (0.94 to 1.31)	1.11 (0.95 to 1.31)	1.11 (0.93 to 1.30)	0.631
Follow-up, mmol/L	1.09 (0.95 to 1.29)	1.05 (0.94 to 1.30)	1.06 (0.94 to 1.24)	0.672
Median change from baseline — %	-0.76 (-12.00 to 12.12)	-0.95 (-8.68 to 12.65)	-0.76 (-12.69 to 12.12)	0.483
**Lp(a), measured**				
Baseline, mg/L	182.68 (77.04 to 397.24)	123.29 (56.49 to 324.39)	204.31 (81.09 to 424.25)	**0.002**
Follow-up, mg/L	154.40 (55.00 to 365.35)	103.20 (51.13 to 218.48)	160.00 (58.06 to 426.89)	0.067
Median change from baseline — %	-4.47 (-39.09 to 29.98)	-6.64 (-39.15 to 33.19)	-3.47 (-39.09 to 29.23)	0.531
**ApoA1, measured**				
Baseline, g/L	1.21 (1.07 to 1.38)	1.24 (1.08 to 1.39)	1.21 (1.07 to 1.37)	0.440
Follow-up, g/L	1.19 (1.03 to 1.39)	1.15 (0.97 to 1.39)	1.18 (1.03 to 1.33)	0.492
Median change from baseline — %	-1.89 (-12.92 to 11.40)	-0.92 (-11.01 to 8.63)	-2.10 (-13.06 to 11.60)	0.729
**ApoB, measured**				
Baseline, g/L	0.73 (0.59 to 0.88)	0.59 (0.49 to 0.78)	0.75 (0.62 to 0.92)	**< 0.001**
Follow-up, g/L	0.62 (0.50 to 0.76)	0.60 (0.49 to 0.72)	0.60 (0.50 to 0.73)	0.882
Median change from baseline — %	-15.83 (-32.48 to 2.72)	-3.17 (-16.95 to 17.50)	-19.35 (-34.56 to 0.00)	**< 0.001**

*Note* LLT, lipid-lowering therapy; LDL-C, low-density lipoprotein cholesterol; RC, remnant cholesterol; HDL-C, high-density lipoprotein cholesterol; TG, triglyceride; TC, total cholesterol; Lp(a), lipoprotein(a); ApoA1, apolipoprotein A1; ApoB, apolipoprotein B

### Changes in lipid profile parameters

At baseline, LDL-C (1.72 mmol/L vs. 2.22 mmol/L, *P* < 0.001), RC (0.46 mmol/L vs. 0.52 mmol/L, *P* < 0.001), nonHDL-C (2.14 mmol/L vs. 2.79 mmol/L, *P* < 0.001), TG (1.29 mmol/L vs. 1.51 mmol/L, *P* = 0.005), TC (3.39 mmol/L vs. 3.95 mmol/L, *P* < 0.001), Lp(a) (123.29 mg/L vs. 204.31 mg/L, *P* = 0.002), and apoB (0.59 g/L vs. 0.75 g/L, *P* < 0.001) levels were significantly lower in the continued low-/moderate-intensity LLT group than in the high-intensity LLT group (Table 2). However, after a median of 3 months’ follow-up, LDL-C was significantly lower in the high-intensity LLT group than in the low-/moderate-intensity group (1.60 mmol/L vs. 1.79 mmol/L, *P* = 0.016). Furthermore, relative changes from baseline in LDL-C (-27.40% vs. -1.56%, *P* < 0.001), nonHDL-C (-25.12% vs. 0.00%, *P* < 0.001), RC (-20.51% vs. -3.90%, *P* = 0.025), TG (-17.82% vs. -6.62%, *P* < 0.001), TC (-18.14% vs. 0.08, *P* < 0.001), and apoB (-19.35% vs. -3.17%, *P* < 0.001) were statistically significant lower in the high-intensity LLT group compared to the continued low-/moderate-intensity LLT group.

### Correlations of percentage changes in LDL-C with RC, nonHDL-C, TG, and apoB

**Fig. 2:**
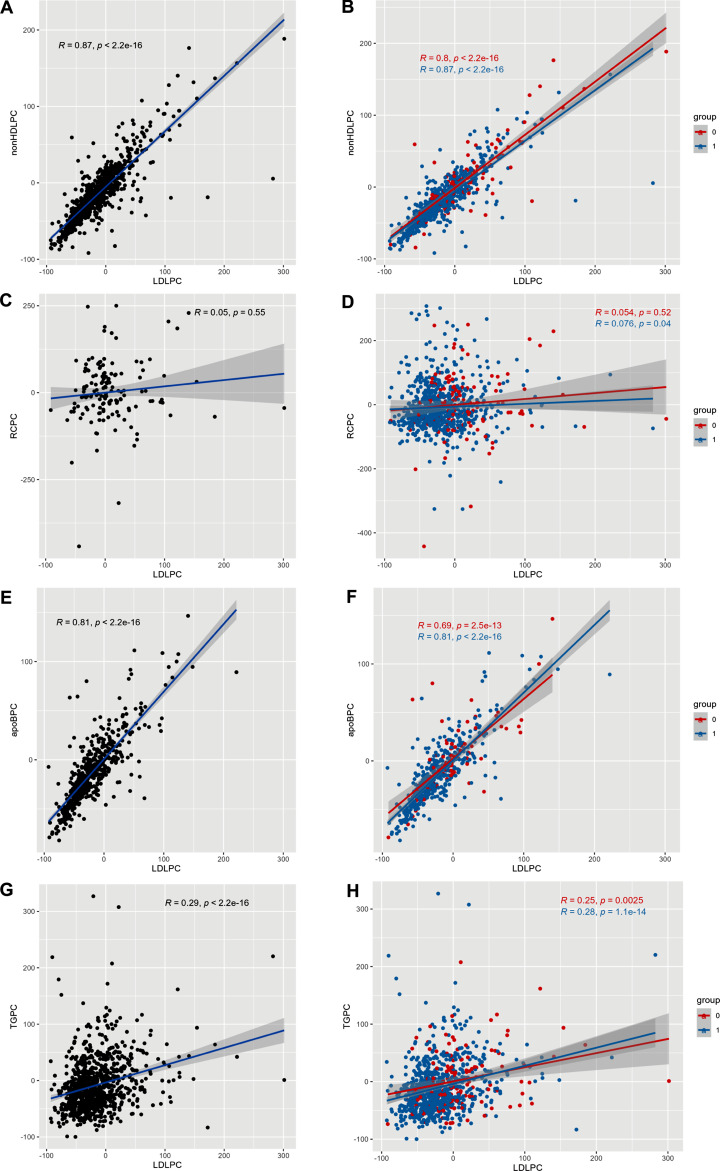
Correlation of percent changes in LDL-C with nonHDL-C, RC, apoB, and TG. The X-axis represents percent changes in LDL-C from baseline to a median of 3 months’ follow-up in all LLT patients (black dots), low-/moderate-intensity LLT (red dots, group = 0), and high-intensity LLT (blue dots, group = 1); the Y-axis represents changes in nonHDL-C, RC, apoB, or TG from baseline to the follow-up in the same respective LLT patients. All correlation coefficients and *P* values are shown in the graph. *Note*: LDLPC, low-density lipoprotein cholesterol percent change; nonHDLPC, nonhigh-density lipoprotein cholesterol percent change; RCPC, remnant cholesterol percent change; apoBPC, apolipoprotein B percent change; TGPC, triglycerides percent change

Percentage changes in LDL-C during follow-up were strongly correlated with percentage changes in nonHDL-C (overall *r* = 0.87, *P* < 0.0001) and apoB (overall *r* = 0.81, *P* < 0.0001) (Fig. 2A and E), and the strong correlation between changes in LDL-C and nonHDL-C existed in two LLT subgroups: the low-/moderate-intensity LLT group (*r* = 0.80, *P* < 0.0001) and the high-intensity LLT group (*r* = 0.87, *P* < 0.0001) (Fig. 2B). However, for the percentage changes in LDL-C and apoB, this strong correlation was only present in the high-intensity group (*r* = 0.81, *P* < 0.0001); in the low-/moderate-intensity LLT group (*r* = 0.69, *P* < 0.0001), it was merely a moderate correlation (Fig. 2F). Changes in LDL-C were also weakly correlated with changes in TG (overall *r* = 0.29, *P* < 0.0001), and this persisted in subgroups (*r* = 0.25 in low-/moderate-intensity LLT group, *P* = 0.0025 and *r* = 0.28 in high-intensity LLT group, *P* < 0.0001) (Fig. 2G and H). In addition, statistically significant correlations between changes in LDL-C and RC were also found (overall *r* = 0.097, *P* = 0.004), but these correlations were only observed in the high-intensity LLT group (*r* = 0.076, *P* = 0.04) and can be considered negligible (Fig. 2C and D).

### Relationships between LLT groups and goal attainment rates for RC and nonHDL-C

**Fig. 3:**
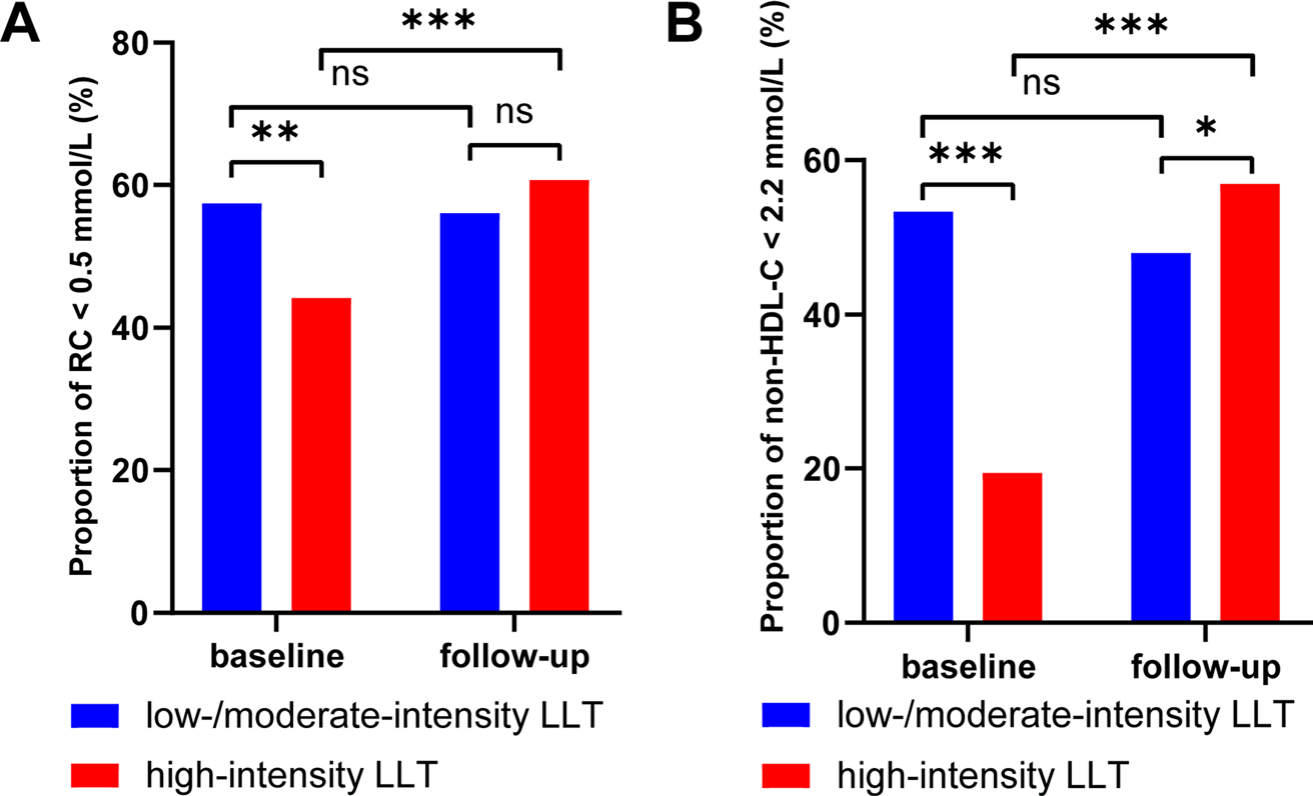
Percentage of patients who achieved RC < 0.5 mmol/L or nonHDL-C < 2.2 mmol/L with LLT. Values shown are calculated RC and nonHDL-C based on measured lipid profiles from baseline and follow-up. The goal attainment differences in the high-intensity LLT group between baseline and follow-up were statistically significant at *P* < 0.001. The treatment difference between low-/moderate-intensity LLT and high-intensity LLT for RC < 0.5 mmol/L goal was not statistically significant. For the nonHDL-C < 2.2 mmol/L goal, high-intensity LLT was better than low-/moderate-intensity LLT at *P* < 0.05. *Note*: LLT, lipid-lowering therapy; RC, remnant cholesterol; nonHDL-C, nonhigh-density lipoprotein cholesterol; ns, not statistically significant; *indicated *P* < 0.05; **indicated *P* < 0.01; ***indicated *P* < 0.001

In the low-/moderate-intensity group, few changes were observed in the goal attainment rates of RC (χ² = 0.12, *P* = 0.725) and nonHDL-C (χ² = 0.66, *P* = 0.416) during follow-up. In the high-intensity group, significant improvements in goal attainment rates for RC (from 44.2% to 60.7%, χ² = 39.23, *P* < 0.001) and nonHDL-C (from 19.4% to 56.9%, χ² = 226.06, *P* < 0.001) were observed after follow-up, and nonHDL-C goal attainment was even better in the high-intensity LLT group (χ² = 3.73, *P* = 0.05) (Fig. 3). In the sensitivity analysis, significant improvements in achieving target levels for both RC and nonHDL-C were observed in the context of combination therapy involving statins and ezetimibe (*N* = 718), the use of PCSK9i (*N* = 30), and the combination of statins, ezetimibe, and PCSK9i (*N* = 26). These findings are consistent with our primary study outcomes (Supplemental Fig. 1).

**Fig. 4:**
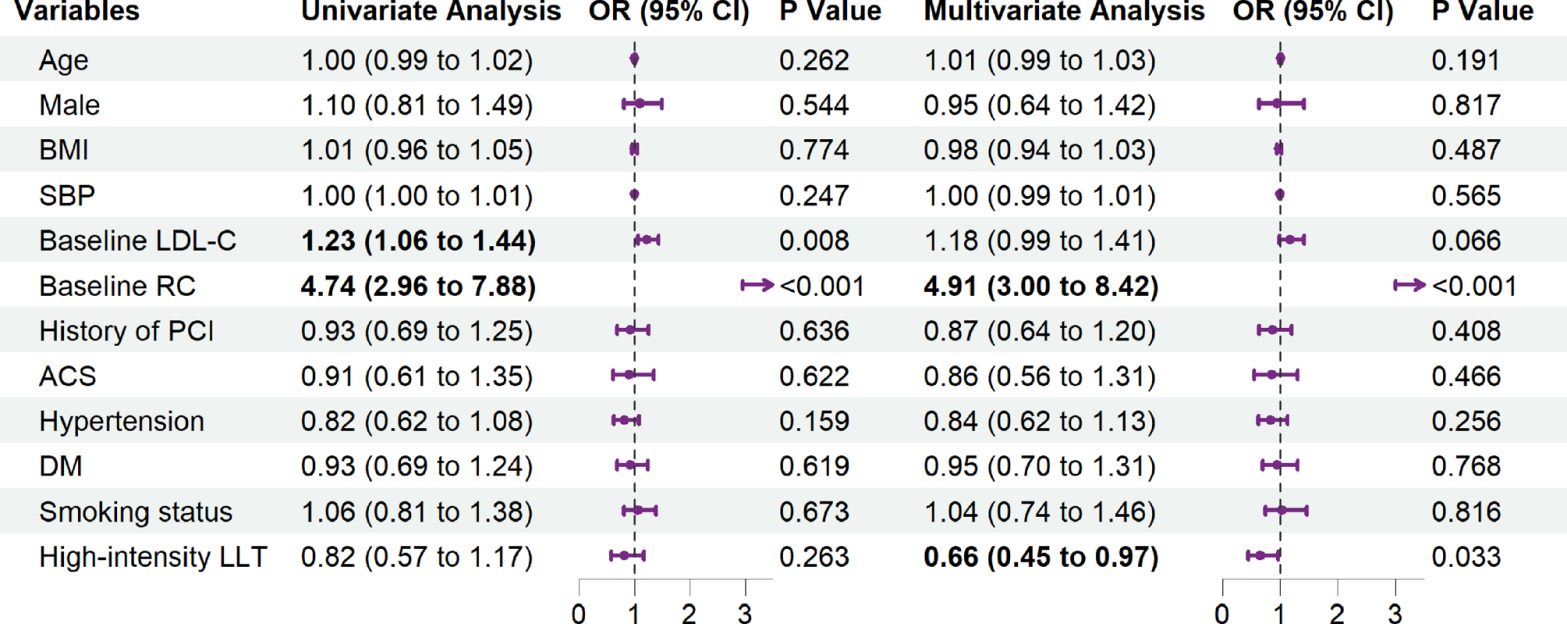
Factors impacting achievement of the RC < 0.5 mmol/L goal for patients treated with LLT. *Note*: BMI, body mass index; SBP, systolic blood pressure; LDL-C, low-density lipoprotein cholesterol; RC, remnant cholesterol; PCI, percutaneous coronary intervention; ACS, admission for acute coronary syndrome; DM, diabetes mellitus; LLT, lipid-lowering therapy

**Fig. 5:**
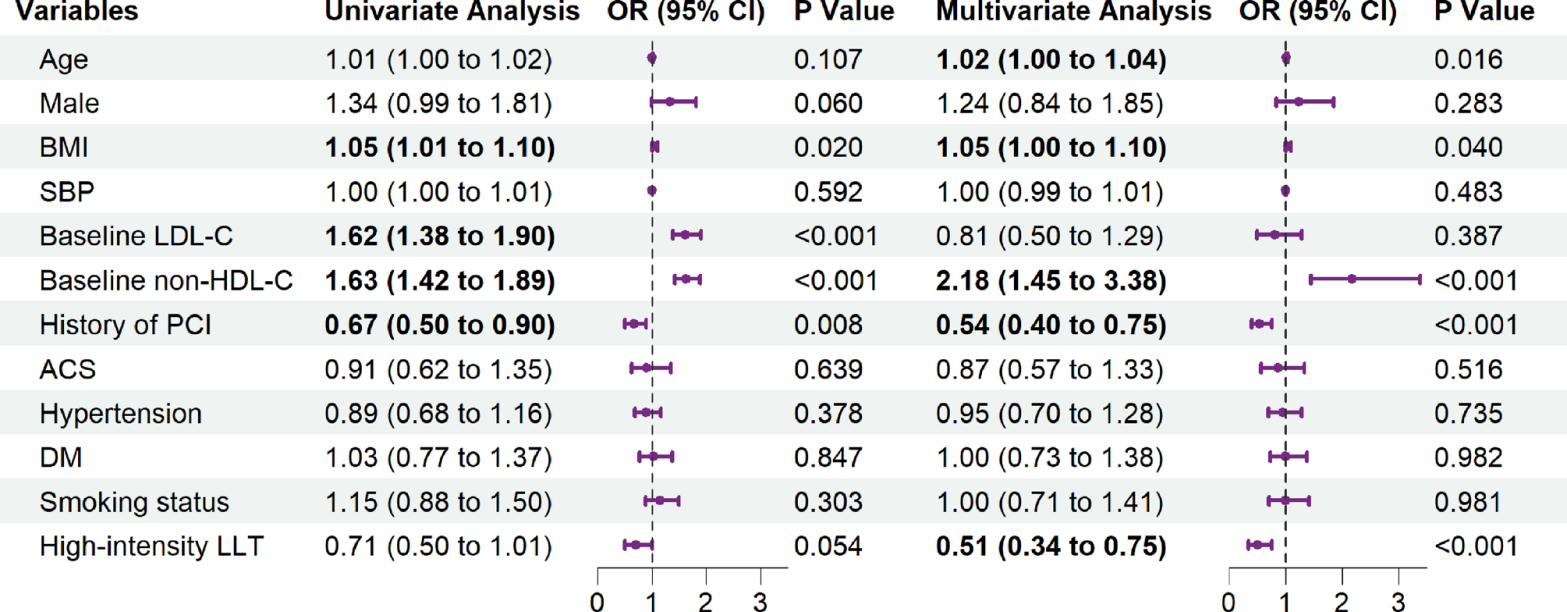
Factors impacting achievement of the nonHDL-C < 2.2 mmol/L goal for patients treated with LLT. *Note*: BMI, body mass index; SBP, systolic blood pressure; LDL-C, low-density lipoprotein cholesterol; nonHDL-C, nonhigh-density lipoprotein cholesterol; PCI, percutaneous coronary intervention; ACS, admission for acute coronary syndrome; DM, diabetes mellitus; LLT, lipid-lowering therapy

After adjusting for certain covariates, the logistic regression results showed that high-intensity LLT could improve the goal attainment rates of both RC [odds ratio (OR) = 0.66; 95% confidence interval (CI), 0.45–0.97; *P* = 0.033] (Fig. 4) and nonHDL-C (OR = 0.51; 95% CI, 0.34–0.75; *P* < 0.001) (Fig. 5). The interaction analysis results indicated that for the RC goal attainment, there was an interaction effect between age and group (*P* for interaction = 0.035). In the age < 60 subgroup, high-intensity LLT was associated with a reduced risk of RC (OR = 0.43; 95% CI, 0.24–0.78; *P* = 0.005). However, in the age ≥ 60 subgroup, although the direction of the effect remained consistent, statistical significance was no longer observed. Regarding the nonHDL-C goal attainment, no significant interaction effect was found between sex and age with LLT Supplementary Table 2).

Safety analysis showed that different LLT groups had no obvious damage to liver and renal function except for ALT (23.00 mmol/L vs. 26.15 mmol/L, *P* = 0.018), nor was there any significant difference in the occurrence of adverse hepatic events (Table 3).

**Table 3:** Safety of LLT with respect to liver and renal function

Variables	Low-/moderate-intensity LLT (*N* = 148)	High-intensity LLT (*N* = 749)		P value
**ALT**				
Baseline, IU/L	23.00 (16.00 to 33.50)	24.00 (16.00 to 34.00)		0.560
Follow-up, IU/L	23.00 (17.41 to 31.20)	26.15 (19.00 to 37.00)		**0.018**
**AST**				
Baseline, IU/L	23.00 (18.00 to 29.00)	24.00 (18.00 to 31.00)		0.300
Follow-up, IU/L	21.25 (17.00 to 27.90)	23.00 (19.00 to 28.00)		0.064
**ALP**				
Baseline, IU/L	70.00 (58.00 to 85.00)	74.00 (61.00 to 87.00)		0.064
Follow-up, IU/L	69.70 (58.90 to 87.25)	73.00 (60.00 to 87.00)		0.488
**TBIL**				
Baseline, µmol/L	10.19 (7.74 to 13.43)	9.65 (7.10 to 12.91)		0.110
Follow-up, µmol/L	12.30 (9.90 to 16.16)	12.29 (9.70 to 16.71)		0.831
**DBIL**				
Baseline, µmol/L	3.39 (2.51 to 4.46)	2.85 (2.07 to 4.05)		**< 0.001**
Follow-up, µmol/L	4.00 (2.83 to 5.55)	4.10 (2.91 to 5.60)		0.384
**Glucose**				
Baseline, mmol/L	6.22 (5.46 to 7.52)	6.09 (5.36 to 7.66)		0.661
Follow-up, mmol/L	6.04 (5.46 to 6.99)	5.92 (5.32 to 6.76)		0.376
**Scr**				
Baseline, µmol/L	82.88 (74.58 to 90.23)	85.47 (76.94 to 95.35)		**0.011**
Follow-up, µmol/L	76.00 (65.70 to 87.00)	76.18 (67.00 to 87.68)		0.641
**Bun**				
Baseline, mmol/L	5.59 (4.65 to 6.54)	5.75 (4.83 to 6.80)		0.188
Follow-up, mmol/L	5.50 (4.70 to 6.37)	5.50 (4.57 to 6.58)		0.874
**UA**				
Baseline, µmol/L	345.11 (296.34 to 417.32)	353.46 (299.03 to 407.36)		0.903
Follow-up, µmol/L	336.00 (291.50 to 402.69)	334.00 (279.50 to 394.78)		0.268
**CK**				
Baseline, IU/L	87.00 (66.00 to 118.50)	85.00 (64.00 to 120.00)		0.810
Follow-up, IU/L	86.80 (65.50 to 121.00)	94.28 (69.45 to 130.35)		0.322
**Any hepatic adverse event**	0 (0%)	5 (0.9%)		0.642
AST > 3×ULN	0 (0%)	3 (0.5%)		0.958
ALT > 3×ULN	0 (0%)	3 (0.5%)		0.981

*Note* ALT, alanine transaminase; AST, aspartate aminotransferase; ALP, alkaline phosphatase; TBIL, total bilirubin; DBIL, direct bilirubin; Scr, serum creatinine; Bun, blood urea nitrogen; UA, uric acid; CK, creatine kinase; ULN, upper limit of normal

## Discussion

RC and nonHDL-C are considered to be important lipid treatment targets in the current recommendations of American and European guidelines along with LDL-C. Research has reported that the shift in lipid-associated risk, characterized by nonoptimal cholesterol, has transitioned from a distinct characteristic of high-income countries, to now one of countries in east and southeast Asia and Oceania as well over the past 40 years. In 2017, nonHDL-C was implicated in an estimated 3.9 million global fatalities, with half of these occurring in the eastern and southern parts of Asia [17]. However, current clinical LLT still primarily focuses on LDL-C reduction. At a time when more and more effective LDL-C-lowering drugs are available, exploring the impact of high-intensity LLT and continued low-/moderate-intensity LLT on RC, nonHDL-C, and other potential targets is important.

Current high-intensity LDL-C-centric treatment was also found to be able to reduce other lipid treatment targets. Compared to the continued low-/moderate-intensity LLT group, the decrease in nonHDL-C, RC, apoB and TG were more significant in the high-intensity LLT group. Additionally, the decrease in LDL-C was correlated with that in nonHDL-C, apoB and TG, but the correlation was stronger with nonHDL-C. More importantly, high-intensity LLT can significantly improve nonHDL-C and RC goal attainment without impairment of liver or renal function.

LDL-C is traditionally recommended as the primary lipid target for treatment in dyslipidemia management guidelines because of its causal association with ASCVD risk. Therefore, the population selected in this study represents patients receiving the most common clinically prescribed LLT, including statins, ezetimibe, and PCSK9 inhibitors that mainly target LDL-C.

However, a growing body of research has suggested that LDL-C in isolation may not be a good measure of ASCVD risk. LDL-C variability is associated with an increased risk of all-cause mortality and cardiovascular hospitalizations, supporting the importance of maintaining a continuous downward trend in LDL-C levels for cardiovascular benefit [18]. Statin therapy significantly reduces major adverse cardiovascular events (MACE) by lowering TC and LDL-C, but a residual cardiovascular risk remains. A meta-analysis covering over 240,000 subjects showed that LLT reduced the risk of MACE by 26% and 15% per 1 mmol/L reduction in LDL-C for patients aged ≥ 75 years and those younger than 75, respectively, implying that there was still a residual relative risk of MACE of 74% and 85% in each group [19]. Even intensive statin therapy (atorvastatin 80 mg/d or pitavastatin 4 mg/d) can reduce the relative risk of coronary events by about 20%, but the residual risk of coronary events remains high [20, 21]. In RCTs comparing high-intensity statin treatment to standard-dose statin treatment, it was found that a significant proportion, ranging from 78 to 87%, of patients in the high-intensity statin groups still exhibited residual CVD risk [22].

Additional pro-atherogenic lipid parameters, such as RC, nonHDL-C, apoB, and Lp(a), provide important predictive information for ASCVD risk assessment and management [23]. In particular, a meta-analysis found that in statin-treated patients, treatment levels of LDL-C, nonHDL-C, and apoB were all associated with the risk of future MACE, but the association was significantly stronger for nonHDL-C than for LDL- C (*P* = 0.002) and apoB (*P* = 0.02) [13]. A 22-year follow-up cohort study in Israel also concluded that nonHDL-C was a better predictor of CVD and all-cause mortality in men than LDL-C [24]. Large cohort studies have also shown that elevated RC increases the risk of MI, ischemic stroke, and PAD. In the Copenhagen General Population Study, for example, elevated RC levels [≥ 1.5 mmol/L (58 mg/dL)] were associated with a higher risk of MI [hazard ratios (HR) = 4.2], ischemic stroke (HR = 1.8), and PAD (HR = 4.8). Similarly, the City of Copenhagen Heart Study also identified a correlation between elevated RC levels and an increased risk of the aforementioned three diseases, with HR of 2.6, 2.1, and 4.9, respectively [10].

Lipid parameters play a crucial role in cardiovascular risk management, as highlighted in various guidelines. For instance, the National Lipid Association recommends targeting nonHDL-C along with LDL-C and designates apoB as only a secondary target [25]. In contrast, European guidelines categorize both nonHDL-C and apoB as secondary targets [1, 26]. Furthermore, although the 2013 ACC/AHA guidelines lack specific nonHDL-C thresholds, recent updates such as the 2016 ACC Consensus Decision Pathway include them for high-risk patients [27, 28]. These evolving guidelines reflect a shift towards a more holistic approach to lipid management, with tailored interventions for different patient populations, including those with diabetes and the elderly, in order to optimize CVD prevention [29].

This study revealed that despite patients transitioning to the high-intensity LLT having higher baseline LDL-C and worse lipid-related targets, after short-term treatment not only did their LDL-C significantly improve, but their atherogenic lipid parameters also significantly decreased, reaching levels similar to those in the low-/moderate-intensity LLT group. Subgroup analysis demonstrated that the combined use of statins with ezetimibe or PCSK9i significantly improved RC and nonHDL-C goal attainment rates. Remarkably, patients on PCSK9i, who initially had lower goal attainment rates for RC and nonHDL-C than those on statin-ezetimibe therapy, showed significant improvements in these parameters at three months. The nonHDL-C goal attainment with PCSK9i surpassed that of continuous low-/moderate-intensity LLT, underscoring PCSK9i’s potential in lipid management optimization. This suggests that transitioning to intensive LLT can further reduce residual risk indicators, which is consistent with many previous studies. For example, one randomized crossover trial of mixed hyperlipidemic patients found that pravastatin, simvastatin, and atorvastatin significantly decreased nonHDL-C levels by 21%, 29%, and 32%, respectively. RC levels were decreased by simvastatin (6%) and atorvastatin (25.9%) significantly, but not by pravastatin (2.9%) [30]. A post hoc analysis of the STELLAR trial also found that both full-dose atorvastatin and rosuvastatin caused significant decreases in TG (− 33.0%, − 27.6%), RC (− 58.7%, − 61.5%), and apoB-48 (− 37.5%, − 32.1%) levels over a 6-week period compared to baseline by similar amounts [31]. Moreover, reports from KISHIMEN Investigators showed that pitavastatin significantly decreased RC levels by 22.8% and also reduced TG levels [32]. Likewise, the PREVAIL US Trial demonstrated that both pitavastatin and pravastatin were capable of effectively lowering the RC levels in patients with dyslipidemias, with pitavastatin exhibiting a more potent effect than pravastatin [33]. More recently, in a post hoc analysis that evaluated data from five randomized controlled trials (RCT), ezetimibe + statins resulted in greater reductions in RC compared to statin monotherapy in both statin-naïve and statin-taking patients [34]. The multicenter LIPID-REAL registry study, involving 652 patients treated with PCSK9 inhibitors (evolocumab or alirocumab), showed significant reductions in RC (from 29.88 mg/dL to 27.30 mg/dL), TC/HDL ratio, TG/HDL ratio, and the TG-to-glucose index at a median follow-up of 187.5 days, with more pronounced decreases observed in patients whose baseline RC exceeded 30 mg/dL [35].

Age and sex play a significant role in the study of associations between nonHDL-C and residual CVD risk as well as all-cause mortality [36]. The findings of this study indicate that sex does not influence the effectiveness of high-intensity LLT, while age may represent a significant factor affecting the control of RC with high-intensity LLT. In younger individuals, high-intensity LLT demonstrates superior RC control compared to low-/moderate-intensity LLT, whereas in older individuals, there is no significant difference in RC control between high-intensity and low-/moderate-intensity therapy. A prospective study involving 95,663 participants with a median follow-up of 11 years found that elevated RC (≥ 1.50 mmol/L) were strongly associated with a higher risk of CVD, and this association was particularly pronounced in young adults (HR = 2.24) [37]. Therefore, high-intensity LLT may provide greater cardiovascular benefits for younger patients. Nevertheless, in the context of the nonHDL-C goal attainment, age does not impact the lipid-lowering efficacy of high-intensity LLT. Thus, from the perspective of nonHDL-C benefits, it is still advisable to consider high-intensity LLT for elderly individuals.

However, despite improvements in residual risk targets such as RC after transitioning to high-intensity LLT, there is still substantial room for further reduction, and this may necessitate the concurrent use of additional medications that lower RC and nonHDL-C. Several studies have shown that fibrates can reduce RC levels. Randomized controlled trials have shown that fenofibrate not only lowers TG but also reduces RC and increases HDL-C in hyperlipidemic subjects as well as type-2 diabetic patients [38–40]. Recently, the novel peroxisome proliferator-activated receptor (PPAR)-α agonist pemafibrate has been found to regulate PPARα expression through its selective affinity for PPARα receptors, which effectively decreases plasma levels of RC and TG, either as monotherapy or in combination with statins [41, 42]. However, the findings from the large-scale phase III PROMINENT trial indicated that for individuals with high TG and diabetes, pemafibrate did not result in a decreased risk of cardiovascular events or mortality even though it led to a reduction of approximately 20–30% each in TG, RC, VLDL-C, and apoCIII [43].

Omega-3 polyunsaturated fatty acids (PUFAs), primarily eicosapentaenoic acid (EPA) and docosahexaenoic acid (DHA), have been shown to be effective in lowering TG and RC and have been proposed as a supplement for cardiovascular health [44]. A pilot study found high-dose EPA/DHA supplementation significantly lowered RC by approximately 3.25 mg/dL and improved the ankle-brachial index in hemodialysis patients with dyslipidemia [45]. In the REDUCE-IT trial, 8,179 statin-treated patients with CVD/diabetes were randomized to receive 4 g/day of icosapent ethyl (IPE) or placebo, resulting in a relative risk reduction in MACE by up to 25% on top of statins [46]. Furthermore, a meta-analysis suggested that there is a cardiovascular benefit from PUFAs containing EPA and DHA, although less pronounced than that observed with IPE [47]. A recent RCT, RESPECTEPA study, also indicated that adding highly purified EPA (1.8 g/d) to statin treatment in stable CAD patients reduced MACE (*P* = 0.055), achieving significance in the composite risk of coronary artery events (*P* = 0.031) [48]. These findings underscore RC as a promising therapeutic target and emphasize the necessity for future RCTs to evaluate the impact of lowering residual-risk-related lipid parameters in patients with ASCVD [49].

Finally, this study did not observe a significant effect of high-intensity LLT on Lp(a), HDL-C or apoA1 levels, nor did it reveal any notable adverse effects on hepatic or renal functions. Importantly, statin intolerance, which is relatively common in clinical settings, necessitates careful management of LLT intolerances [50]. In the current era, the expansion of non-statin alternatives, including PCSK9 inhibitors, bempedoic acid, and inclisiran, offers enhanced strategies for the improved management of vulnerable patients [51, 52]. This finding underscores the complexity of lipid management in CVD and highlights the necessity for targeted interventions and personalized treatment based on the patient’s specific CVD risk factors [53].

## Study strengths and limitations

This study revealed the effectiveness of current clinical high-intensity LLT in broader lipid management beyond mere LDL-C reduction, with significant implications for improving goal attainment of lipid-related residual risk factors in lipid therapy and for guiding future treatment strategies in real-world CVD management. Limitations of this study include its single-center nature, relatively small sample size, short-term follow-up (median of 3 months), use of calculated RC, and lack of data on MACEs. The accuracy of calculated RC can be compromised when it is derived from calculated LDL-C rather than directly-measured LDL-C, as the former approach does not quantify cholesterol levels within specific lipoprotein classes or subfractions [54]. However, calculated LDL-C is favored for its cost-effectiveness and simplicity that allows for straightforward computation using conventional lipid profiles. Subsequent studies need to employ larger, more diverse samples and lengthen follow-up periods to validate and expand upon these findings.

## Conclusion

Current clinical LLT, which focuses primarily on reducing LDL-C, also lowers the levels of RC, nonHDL-C, apoB, and TG, which are crucial in managing residual lipid risk in CVD patients. The decrease in these lipid-related residual risk factors was more significant in the high-intensity LLT group compared to the continued low-/moderate-intensity LLT group in this study. High-intensity LLT significantly improved nonHDL-C and RC goal attainment, along with demonstrating good safety, which is critical for optimal CVD management and therapy adherence. More focused treatment strategies that target RC might be necessary to further reduce residual lipid risk, thus providing a potential direction for future clinical research.

### Electronic supplementary material

Below is the link to the electronic supplementary material.

Supplementary Material 1

## Data Availability

As a result of reasons of sensitivity, the data used to support the findings are not publicly available.

## Abbreviations

ACS: Acute coronary syndrome
ALT: Alanine aminotransferase
ApoB: Apolipoprotein B
ASCVD: Atherosclerotic cardiovascular disease
AST: Aspartate aminotransferase
BMI: Body mass index
DBP: Diastolic blood pressure
DM: Diabetes mellitus
HDL-C: High-density lipoprotein cholesterol
LDL-C: Low-density lipoprotein cholesterol
LLT: Lipid-lowering therapy
Lp(a): Lipoprotein(a)
MACE: Major adverse cardiovascular events
MI: Myocardial infarction
NonHDL-C: Nonhigh-density lipoprotein cholesterol
PAD: Peripheral artery disease
PCI: Percutaneous coronary intervention
RCT: Randomized controlled trials
RC: Remnant cholesterol
SBP: Systolic blood pressure
TC: Total cholesterol
TG: Triglyceride
VLDL: Very low-density lipoprotein
