# Supplementary material for: The effect of lipid-lowering therapy on lipid-related residual risk factors: a prospective study
Source: Lipids Health Dis. 2024 May 7;23:134. doi: 10.1186/s12944-024-02078-0 (PMC11075277; doi:10.1186/s12944-024-02078-0)
Supplement: Supplementary file 1 — Supplementary Material 1 [file 12944_2024_2078_MOESM1_ESM.docx]

**Supplemental Table 1. Detailed drug use in two groups**

| **Medication** | **low-/moderate-intensity LLT (N=148)** | **Medication** | **high-intensity LLT (N=749)** |
| --- | --- | --- | --- |
| Atorvastatin | 59 (39.86%) | Statin+Ezetimibe, n (%) | 718 (95.86%) |
| 10mg, n (%) | 1 (0.68%) |  |  |
| 20mg, n (%) | 58 (39.19%) |  |  |
| Rosuvastatin 10mg, n (%) | 59 (39.86%) | Statin+PCSK9i, n (%) | 3 (0.40%) |
| Pravastatin 40mg, n (%) | 7 (4.73%) | Ezetimibe+PCSK9i,  n (%) | 1 (0.13%) |
| Fluvastatin 80mg, n (%) | 3 (2.03%) |  |  |
| Pitavastatin | 16 (10.81%) | Statin+Ezetimibe+  PCSK9i, n (%) | 26 (3.47%) |
| 2mg, n (%) | 7 (4.73%) |  |  |
| 4mg, n (%) | 9 (6.08%) | Atorvastatin 40mg,  n (%) | 1 (0.13%) |
| Ezetimibe 10mg, n (%) | 3 (2.03%) |  |  |

All data were shown by n (%). Statin monotherapy was presented by type and dosage. PCSK9i: Proprotein Convertase Subtilisin/Kexin type 9 (PCSK9) inhibitor.

**Supplemental Table 2. The association between LLT and achievement of the RC< 0.5 mmol/L and nonHDL-C < 2.2 mmol/L goal for the total/subgroup population**

|  |  | OR | 95% CI | *P* Value | *P* for interaction |
| --- | --- | --- | --- | --- | --- |
| RC < 0.5 mmol/L goal | | | | | |
| Age |  | 1.01 | 0.99-1.03 | 0.191 | 0.035 |
| Sex (male) |  | 0.95 | 0.64-1.42 | 0.817 | 0.216 |
| High-intensity LLT | Total | 0.66 | 0.45-0.97 | 0.033 |  |
|  | Age < 60 | 0.43 | 0.24-0.78 | 0.005 |  |
|  | Age ≥ 60 | 0.97 | 0.57-1.65 | 0.906 |  |
| NonHDL-C < 2.2 mmol/L goal | | | | | |
| Age |  | 1.02 | 1.00-1.04 | 0.016 | 0.102 |
| Sex (male) |  | 1.24 | 0.84-1.85 | 0.283 | 0.804 |

Note: LLT, lipid-lowering therapy; RC, remnant cholesterol; nonHDL-C, nonhigh-density lipoprotein cholesterol; OR, odds ratio; CI, confidence intervals.


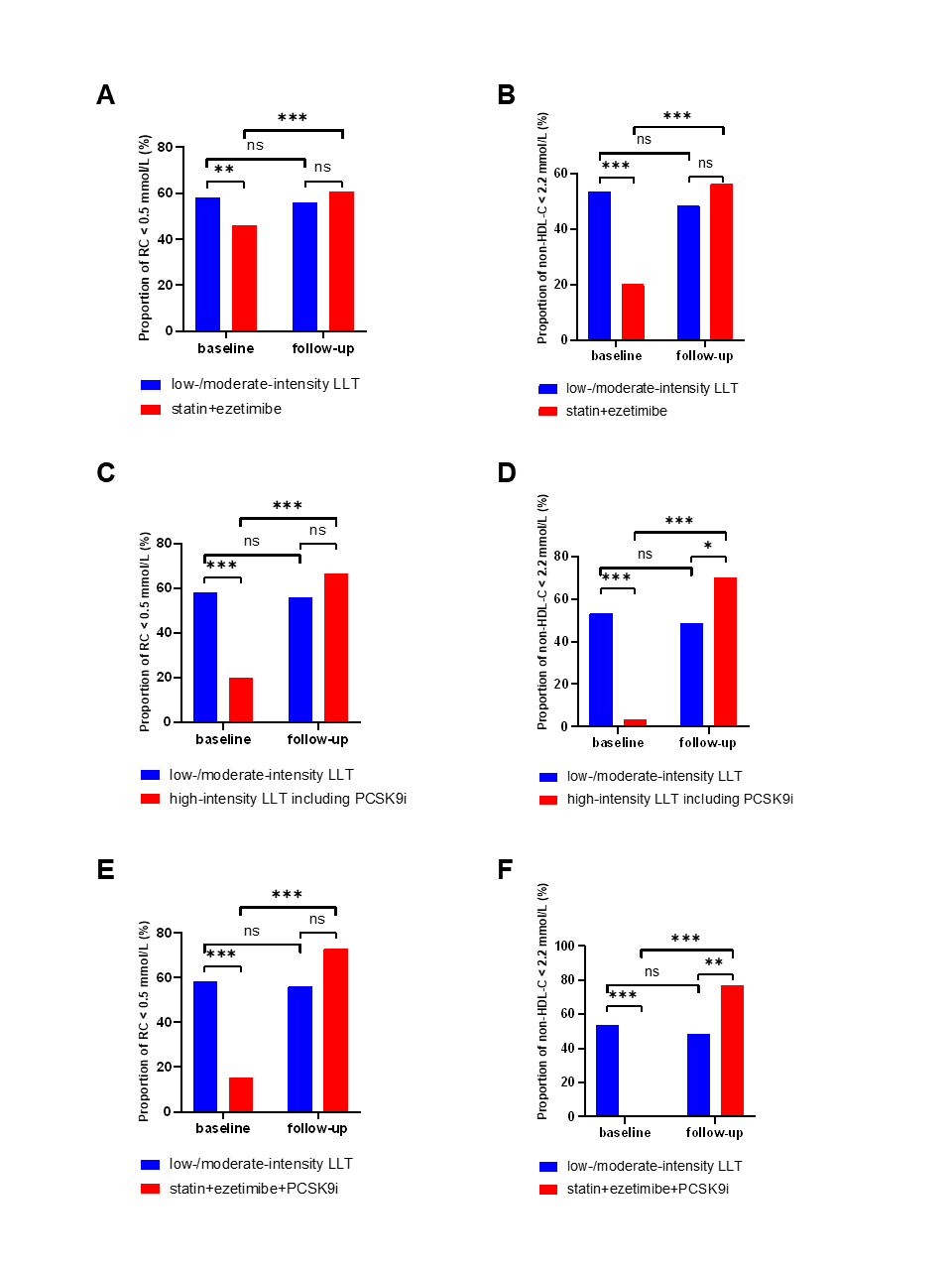


**Supplemental Figure 1. Percentage of patients who achieved RC < 0.5 mmol/L or nonHDL-C < 2.2 mmol/L with LLT**

Values shown are calculated RC and nonHDL-C based on measured lipid profiles from baseline and follow-up. Note: LLT, lipid-lowering therapy; RC, remnant cholesterol; nonHDL-C, nonhigh-density lipoprotein cholesterol; PCSK9i: Proprotein Convertase Subtilisin/Kexin type 9 (PCSK9) inhibitor; ns, not statistically significant; *indicated P < 0.05; **indicated P < 0.01; ***indicated P < 0.001.
